# What condition leads to an unreasonable pharmaceutical price? Impact research on the effect of medical service provision on pharmaceutical price regulation based on fuss-set QCA

**DOI:** 10.1371/journal.pone.0284129

**Published:** 2023-04-13

**Authors:** Lingzhi Brian Fang, Xinmeng Wang, Liu Tang

**Affiliations:** 1 School of Journalism, Fudan University, Shanghai, China; 2 School of Management, Fudan University, Shanghai, China; Sichuan Agricultural University, CHINA

## Abstract

Given that the pharmaceutical market has experienced severe market failures, it is necessary that we regulate pharmaceutical prices for many countries. Toward ensuring that pharmaceutical price regulation is efficient, this study investigated the antecedents that lead to an unreasonable pharmaceutical price. Based on 33 case-study countries, this study utilized QCA to analyze the conditional configuration of unreasonable pharmaceutical prices from the perspective of medical service provision. The results showed that the causes of unreasonable pharmaceutical prices are configured by medical service provision, especially cost compensation systems and payment mechanism. This study’s conclusions contribute to the research on pharmaceutical price regulation and the institution of medical service provision.

## Introduction

Pharmaceutical price regulation has long been a problem for many countries. In China, many people argue that the prices are too high for them to enjoy healthcare services. This is now being addressed by the Chinese government, and corresponding actions are being taken [1,2]. Not only that, China is a typical example to address this administrative issue. In other countries, such as Germany and USA, diversified steps have been adopted for tackling the pharmaceutical price. Yet, different countries have their own situation to address, with potentially varied impacts of pharmaceutical price regulation. Prior research demonstrated that regulating the pharmaceutical price has a negative effect on innovation or cost control [3–5]. Overall, regardless of the attitude toward pharmaceutical price regulation in a specific country, there is still a focus on tackling the problem of unreasonable pharmaceutical prices. Thus, it is of great necessity to detect the antecedents that result in unreasonable pharmaceutical prices.

There are diversified ways to introduce health regulation of pharmaceutical prices. For instance, several related studies revealed that the Chinese government has approached its reform of unreasonable pharmaceutical prices by addressing pharmaceutical affordance, the supply chain, the institution of the healthcare system, etc. [6–8]. Similarly, in other countries around the world, several approaches have been adopted to regulate pharmaceutical prices [9,10]. However, prior studies ignored the role played by medical service provision in contributing to unreasonable pharmaceutical prices, even though plenty of studies attempted to deal with the pricing problem. Therefore, the research gap provides an opportunity to add a new point of view to the literature, which we sought to fill through this study.

Thus, the main research question of this study is clear. We aim to detect the relationship between medical service provision and unreasonable pharmaceutical prices in order to fill the mentioned research gap. When it comes to the methodology, we relied on quantitative comparison analysis (QCA) rather than big data analysis [11]. In comparison to big data analysis, QCA has the advantage of multi-configuration analysis. This contributes to the fitness of this research question. Lastly, this study offers several theoretical contributions and practical implications for academics. Specifically, this study established the relationship between medical service provision and unreasonable pharmaceutical prices in detail. The results of QCA clarify the fact that the cost compensation system, payment mechanism, Tiered healthcare system, and induced demand exerts a great impact on unreasonable pharmaceutical price [12–14]. Consistent with such fact, the practical implications can be provided intothe medical service provision revolution.

The paper is organized as follows. In the first section, the literature regarding related theory and research is reviewed. Based on the literature review, a configuration framework of pharmaceutical prices is proposed. Next, in the second section, QCA is utilized to answer the research question. After analyzing 33 case-study countries, QCA results including a necessity analysis of a single condition and a sufficiency analysis of conditional configurations are provided. At the end of this paper, further discussion is offered. Above all, this study contributes to the research on pharmaceutical price regulation.

## Literature review

In this section, several studies are reviewed. This study introduced the theoretical foundation initially. The introduction of the theoretical foundation offers evidence to support our configuration framework theoretically. After such a precise introduction, the current research on medical service provisionand pharmaceutical prices would be reviewed. According to the prior studies, medical services provision constitutes the services provided by hospitals and physicians, including diagnosis, prevention, and treatment of diseases, delivery, prescription, and other additional services such as hospitalization, emergency care, registration, emergency services, etc. [15,16]. Overall, this part of the paper, therefore, mainly concentrates on the research on medical service provision from the perspective of the hospital and physician. To precisely explore which factors influence the pharmaceutical price, this study relies on the theory of the health economy and regulation economics.

### Theoretical foundation: A necessity to regulate medical service

According to the economics of regulation, there are three main types of regulation [17,18]. The first type is monopoly regulation, which is mainly government regulation of monopolistic industries in the market. According to the first principles of welfare economics, the competitive equilibrium allocation is Pareto efficient. Therefore, if the monopoly price set by the monopolist is not Pareto efficient, there will be a Pareto improvement. At this point, government intervention is needed so that the allocation of resources in the market gradually becomes Pareto efficient, which, in turn, raises the overall welfare of society, as well. The second type is economic regulation. Regulatory economics has a strict definition of economic regulation, which mainly refers to the restriction of firms’ decisions in terms of price, output, entry, and exit. Specifically, this type of regulation is mainly a direct government regulation of the structure of some industries, and the economic performance of a certain industry, mainly with a specific industry as the object of regulation.

Based on the types of regulation, it can be observed that if one industry needs to be regulated, it must have a positive externality, and the market has the characteristics of information asymmetry. Thus, regulation is designed to overcome market failures and thereby redirect the market to a state of Pareto equilibrium. When it comes to the pharmaceutical market, according to prior studies, this market is a main object of regulation [7,10]. The first reason for this is that the market for medical services has strict positive externalities. The government needs to increase its supply and regulate that to prevent it from charging too much. The second point is that the market for medical services is inherently characterized by serious information asymmetry, with medical service providers holding a large amount of professional information and the physician-patient relationship being mostly a principal-agent relationship. This makes the market for medical services vulnerable to entering a state of serious market failure. Accordingly, the government needs to regulate this field to develop in a healthy and orderly direction and enable people to enjoy convenient medical services and improved social welfare.

### Medical service provision

Though we have explored the theoretical foundation of this study and thus revealed the necessity to regulate medical services, there is still a research gap in the research area, resulting in a controversial situation.

According to previous research on medical service provision, few studies explored medical service provision. Plenty of research has focused on its pricing strategy and service affordability [2,13,19]. For example, Yang et al. have explored the relationship between pharmaceutical prices and the supply chain [7]. Not only that, Yip and Hsiao discussed that the medical system in China has been trapped in a dilemma due to the policymakers [20]. Based on this, current studies have attributed the dilemma in the medical system to relatively external factors. Specifically, whether supply chain or policymakers, these factors describe an external environmental impact on unreasonable pharmaceutical prices. Based on this, current studies have ignored the internal systematical factors resulting in unreasonable pharmaceutical prices, thus the research gap has been addressed.

For the most significant part of the internal systematical factors of impact on unreasonable pharmaceutical prices, the medical service provision is inevitable [4,16]. In detail, medical service provision can be categorized into two parts. One part is the medical system, which refers to a whole system of hospitals and the healthcare system provides medical services. The other part is physician, which refers to the doctor, nurse, or other healthcare workers in the medical system for providing medical services. The medical system represents a relatively macro viewpoint for understanding medical service provision, while the physician represents a relatively micro perspective.

#### Medical service provision from the perspective of the medical system

Some pieces of literature reveal that medical services can heavily affect pharmaceutical prices. Support for this effect is twofold. On the one hand, the medical care system exerts a function on the pharmaceutical price. Numerous studies show that different countries have different healthcare systems, which have been established and developed according to their national conditions. For example, in China, the government has revealed a plan for a healthcare system reform to enhance the welfare and popularity of the nation. A tiered medical care system involves stratifying medical services and providing them to the corresponding patients. A tiered medical care system existed before China’s healthcare reform in 1998, but the increasing economic and social development after 1998 and the rising demand for medical services led to the abolition of tiered medical care during China’s healthcare reform in 1998 [20]. Despite this, currently, the General Office of the State Council in China is calling for the gradual re-establishment of a tiered healthcare system. Thus, current studies have begun to discuss how to reform the healthcare system and identify the problems that could be brought about by a tiered healthcare system in China [20,21].

On the other hand, the financial systems of healthcare organizations also have a significant impact on pharmaceutical prices, including payment and cost compensation mechanisms. There are many different types of payment systems for medical services, but in general, they can be divided into two main categories: the first is prepayment, which covers the costs that patients will incur before they enter the hospital to see a doctor; the second is post-payment, which covers these after the patient enters the hospital for medical services. The prepayment systems include prepayment by a global budget, capitation and a diagnosis-related group-prospective payment system (DRGs-PPS); post-payment systems are mainly fee-for-service (FFS), which include payment by service unit, and case-based reimbursement [19,22]. Such payment models affect pharmaceutical prices significantly.

Then, the hospital cost-reimbursement system is concerned through what channels and how the costs of healthcare systems are covered. For a hospital with extremely large positive externalities, it would be very unrealistic to rely solely on the hospital itself to earn profits to compensate for its financial costs. According to prior studies, several factors contribute to this situation. The first point is that the supply of resources in the medical market is much smaller than the demand, and the price of medical services is bound to go up so much that the public cannot enjoy medical services [23]. The second point is that the medical market needs a wide range of innovation, and the high cost of innovation will lead to unattainable prices for medical services if only a compensation system is adopted [24]. As a social welfare benefit to the public, medical services must be made available to the public at affordable prices. Thus, the hospital cost-compensation system can be seen as the government’s disguised delivery of medical services to the public, so as an important part of the medical service delivery model, this has a huge impact on pharmaceutical prices.

#### Medical service provision from the perspective of the physician

In a lot of prior studies, the behavior of physicians in their treatment procedures was investigated. McGuire and Pauly proposed the classical standard model of physician behaviors [25]. This led pharmaceutical scholars to focus on the role physicians play. Specifically, this model is based on maximizing physicians’ utility by describing their behavior. In this model, physicians gain utility through their net income, leisure time, and inefficient inducements. Inefficient inducement by the physician leads the patient to consume more medical services than they need.

When considering the physician behavior model, we can see that this model of behavior overlaps with the classical induced demand theory. Based on the induced demand theory, the standard model of physician behavior precisely describes how pharmaceutical prices linked with the behavior of physicians. When physicians perform induction, they are reluctant to do so for patients who do not have great disposable income. As such, the degree of induction is aversive for physicians. The higher the inducement level, the lower the utility of the physician. Therefore, the lower the physician’s income, the more the physician induces the patient the consume pharmaceuticals [26].

Yet, the reality is constantly challenging what the scholars once believed. Health economists have modeled induced demand to study the behavior of physicians [26–28]. If all physicians induce the same level of demand on their patients, then it appears that there is actually no induced demand. This poses a challenge to health economists. So, health economists study the effect of lower profit margins on physicians’ behavior. As the above-mentioned content shows, physicians induce demand on patients when the profit margins are reduced. This is because doctors need to make up for the loss of profitability by using induced demand to generate additional income.

Although the connection between profits and induced demand has been revealed, this behavior of physicians still depends on the complexity of reality. Nonetheless, it provides us with an appropriate entry point to explore the impact factors on pharmaceutical prices, which facilitates our understanding of how to regulate pharmaceutical prices.

### Configuration framework of the pharmaceutical price

On the basis of mentioned literature review, the research gap can be further detailed. As the previous studies revealing, according to the externalities and public welfare of healthcare system, the medical service possesses information asymmetry, resulting in market failure. Thus, the necessity of regulation on medical services is inevitable. Based on this, regulation economics provides the theoretical foundation of this study.

Moreover, the medical service provision can be understood from the medical system and physician aspects. Specifically, in terms of the medical system, the tiered healthcare system, payment mechanism and cost compensation system are important aspects, accordingly. However, there is an absence of studies into the impact of the unreasonable pharmaceutical prices exerted by these 3 aspects, according to previous studies [4,8,20]. When it comes to the micro perspective, the role of the physician should be not neglected. According to previous studies, the induced demand can reflect the role played by the physician in the medical service provision [27]. Whereas consistent with the lack of studies on the relationship between induced demand and pharmaceutical prices, this study decided to explore such a relationship, in order to fill up the research gap.

**Fig 1:**
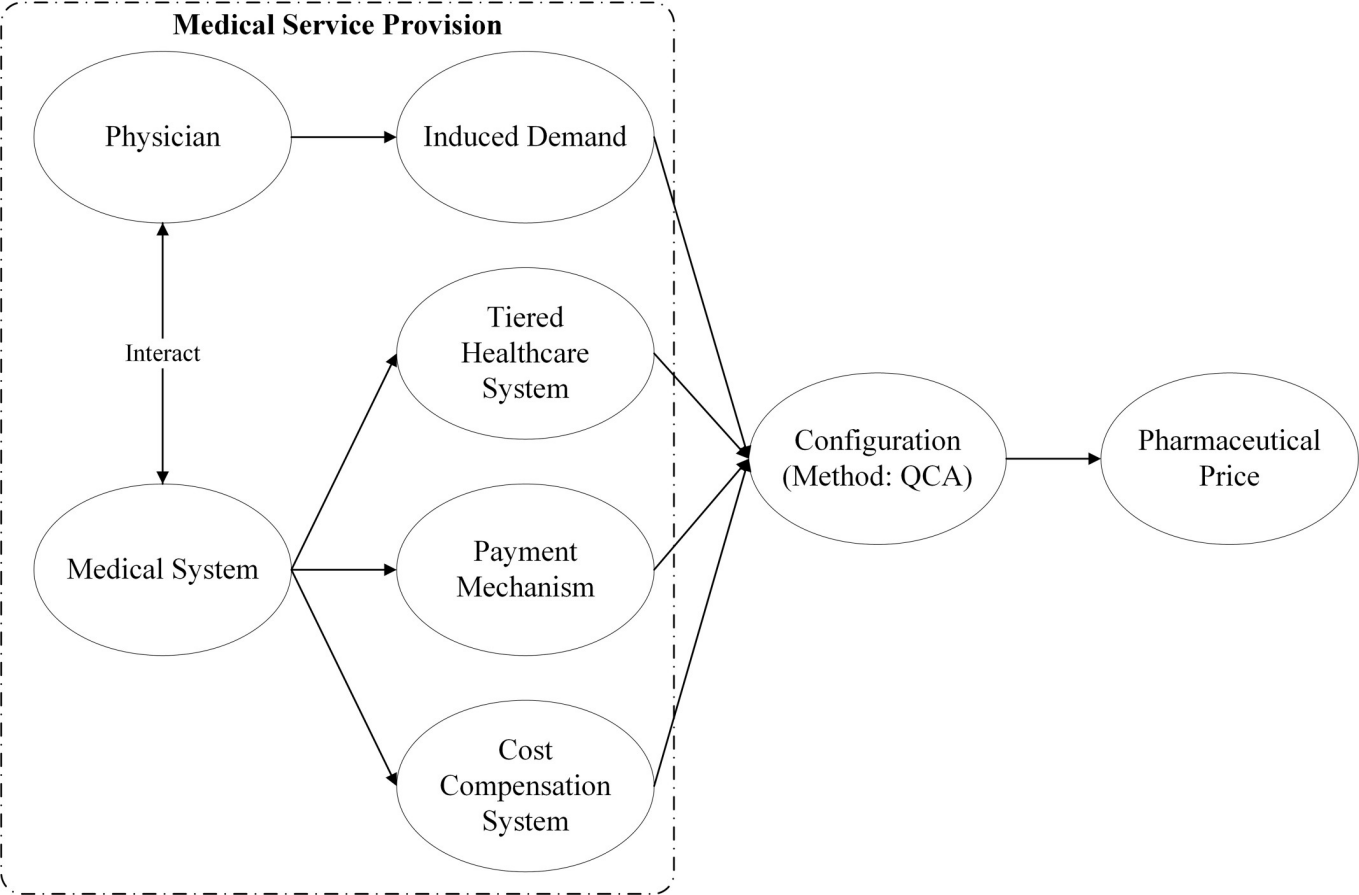
Configuration framework of pharmaceutical price.

Based on such analysis, a configuration framework of pharmaceutical prices is proposed, as detailed in Fig 1.

To explore which factors impact pharmaceutical prices, this study investigated different factors from the perspectives of the physician and medical system. For the physician, induced demand acts as a factor in how they exert a function on pharmaceutical prices. The medical system, tiered healthcare system, payment mechanism and cost-compensation system are mainly considered to affect pharmaceutical prices. Yet, according to the above-mentioned literature review, it is greatly complex how they result in unreasonable pharmaceutical prices. Thus, qualitative comparative analysis (QCA) was adopted as the research method to establish the conditional configuration of antecedents; in this way, multivariate patterns of influence on pharmaceutical prices were revealed.

## Methods

Given the complexity of influencing factors on pharmaceutical prices, the traditional hierarchy regression was not fit for this study. Instead, a new approach was adopted.

Qualitative comparative analysis (QCA) was proposed by Charles Larkin in 1987 [29]. This method combines qualitative and quantitative analyses and is mainly used to analyze the effect of a combination of multiple factors on an outcome [30,31]. It is currently nearly 30 years since this method was proposed, but it is still an emerging empirical method. Although there is considerable room for development and improvement, QCA mainly concentrates on multiple conjunctural causations and is suitable for tackling the research question of this study.

By associating with the advantages of QCA, it is appropriate to adopt this method for this study. QCA has advantages in tackling the complexity of influencing factors according to the prior studies. Not only that, QCA specializes in problems with limited samples. On the basis of the features of QCA, we decided to adopt QCA as the main methodology in this article.

### Sample

For QCA of quantitative data, this study sampled diversified countries to gather data. To ensure the comprehensiveness and diversity of the cases, they were identified through the literature and information websites based on a search of relevant materials. Since there are many case countries, but few case countries with special characteristics, we carefully extracted 33 representative countries based on the available data. These 33 countries cover five continents of the world, with various economic growth conditions. Based on the economic development measured by the GDP per capita in 2021, we categorized these case countries into two types: developed and developing. Table 1 shows the sample scenario.

**Table 1:** Category of the cased countries.

Country category	Cased countries
Developed country	Denmark, Finland, Canada, Czech Republic, France, Germany, Austria, Ireland, Italy, Japan, Norway, Belgium, Portugal, Spain, Sweden, United Kingdom, United States, Australia, Korea, Netherlands, Switzerland
Developing country	China, Kyrgyzstan, Tajikistan, Brazil, Zambia, Mongolia, Poland, Greece, Russia, Tanzania, Slovakia, Hungary

Note: A developed country is judged by whether its GDP per capita is above $20,000.

The sample we have to choose is for ensuring the reliability and validity of this study. these 33 countries reflect the circumstances of the majority of countries all over the world. Thus, the sample we adopted is representative. In accordance with this sample, the reliability and validity of the study can be guaranteed.

### Data collection and processing

#### Data collection

The case data in this paper were mostly obtained from the literature and several reports. The literature and reports mostly came from selected websites, including the official websites of the WHO, OECD, Emmis Health Services, World Bank, etc. To explore the mechanisms of pharmaceutical pricing, we first needed to determine the outcome variable, i.e., whether pharmaceutical prices are unreasonable. This has a relative meaning as there is no absolute measure of whether a pharmaceutical price is unreasonable. In view of this, we proposed using the markup rate of pharmaceutical prices to measure how high or low pharmaceutical prices were. The assigned value on other variables is apparent. We have checked all the reports and documents we have gathered before, in order to figure out the description of the medical service provision from the 4 aspects of induced demand, tiered healthcare system, payment mechanism and cost compensation system. Table 2 displays the pharmaceutical prices for the entire set of case countries.

**Table 2:** Pharmaceutical prices of the cased countries.

Price standard	Cased countries
Pharmaceutical price is unreasonable	China, Kyrgyzstan, Tajikistan, Brazil, Zambia, Mongolia, Poland, Russia, Tanzania
Pharmaceutical price is reasonable	Denmark, Finland, Canada, Czech Republic, France, Germany, Austria, Ireland, Italy, Japan, Norway, Belgium, Portugal, Spain, Sweden, United Kingdom, United States, Australia, Korea, Netherlands, Greece, Slovakia, Hungary, Switzerland

#### Data processing

In this study, the data came from a variety of reports and pieces of literature. To ensure that the QCA was valid, assigning cause and result variables was of great necessity.

In terms of induced demand, we used whether or not the case had a drug-based healthcare system to determine the value assigned. In terms of a tiered healthcare system, the decision was made directly by looking at whether the case country had a tiered healthcare system. In terms of a payment mechanism, we assigned a value to the variable by looking at whether the case country had a DRG system. In terms of cost compensation, we assign a value by looking at whether the public hospital in the case country received more than 50% of the financial compensation. The outcome variable, unreasonable pharmaceutical prices, was assigned by classifying the case countries.

Specifically, the five variables were assigned “0” or “1” by the above process. Specifically, “0” represented an absence of the condition. For example, if “induced demand = 0”, this indicated that the case country had no drug-based healthcare system, i.e., induced demand, and vice versa. Moreover, the pharmaceutical price was named as an outcome variable, with “0” representing a country with reasonable pharmaceutical prices, and “1” representing a country with unreasonable pharmaceutical prices. Table 3 displays the results of data processing.

**Table 3:** True rable of the cased countries.

ID	THS	PM	CCS	Number of countries	Proportion
0	1	1	1	17	51%
0	0	0	0	5	15%
0	1	0	1	4	12%
0	0	1	1	2	6%
0	1	0	0	1	3%
0	0	0	1	1	3%
1	1	1	0	1	3%
1	0	1	1	1	3%

Note: ID represents “induced demand”; THS represents “tiered healthcare system”; PM represents “payment mechanism”; CCS represents “cost compensation system”.

#### Solution coverage and consistency

Consistency and coverage are two concepts based on the logic of qualitative comparative analysis. The formulas for both are as follows:

$$\mathrm{Consistency}\;(Xi\leq Yi)=\sum (\min(X_{i},Y_{i}))/\sum (X_{i})$$ (1)

$$\mathrm{Coverage}\;(Xi\leq Yi)=\sum (\min(X_{i},Y_{i}))/\sum (Y_{i})$$ (2)

Consistency evaluates the relationship between multiple cases given a combination of factors and the study results. Coverage, on the other hand, evaluates the extent to which certain factor combinations quantitatively occupy a ratio of outcome cases.

Consistency varies continuously between 0 and 1, and it mainly describes asymmetric relationships. If the value of consistency is very close to 1, it shows that the results obtained from the analysis describe a perfect asymmetric relationship, and vice versa, an incomplete asymmetric relationship. The coverage also varies continuously between 0 and 1; this describes the uniqueness of the path, i.e., the combination of X factors is the only combination of factors leading to Y results. In describing the set affiliation, when *Consistency* (*Xi Yi*) closer to 1, this indicates that Set A is very likely to be affiliated with Set B. When *Coverage* (*Xi Yi*) is closer to 1, this indicates that set A is very unlikely to be affiliated with set B. In general, we require these two indicators to take values above 0.8.

## Results

### Necessity analysis of the single condition

In most QCA studies, the first step is to conduct a necessity analysis of a single condition. From a set-theoretic perspective, the necessity analysis of a single condition is a test of whether the set of results is a subset of some set of conditions. In fsQCA 3.0, if a condition is always present when the outcome occurs, then that condition is necessary for the outcome [32]. Consistency is an important criterion for a necessary condition, and a condition is considered necessary for an outcome when the consistency level is greater than 0.9 [31,33]. Table 4 displays the results of single condition necessity analysis. Specifically, in Table 4, “~” represents the absence of an antecedent condition.

**Table 4:** Single condition necessity analysis of cased countries.

Antecedent conditions	Unreasonable pharmaceutical price			Reasonable pharmaceutical price		
	Consistency	Coverage	Combined	Consistency	Coverage	Combined
ID	0.667	0.222	0.279	0.333	0.042	0.020
~ID	0.233	0.778	0.088	0.767	0.958	0.825
THS	0.130	0.333	0.058	0.870	0.833	0.876
~THS	0.600	0.667	0.316	0.400	0.167	0.058
PM	0.095	0.222	0.047	0.905	0.792	0.872
~PM	0.583	0.778	0.306	0.417	0.208	0.065
CCS	0.040	0.111	0.033	0.960	1.000	0.995
~CCS	1.000	0.889	0.938	0.000	0.000	0.000

Note: ID represents “induced demand”; THS represents “tiered healthcare system”; PM represents “payment mechanism”; CCS represents “cost compensation system”.

According to the results provided by Table 4, it can be observed that “~CCS” is a necessary condition that leads to unreasonable pharmaceutical prices. Specifically, if the cost compensation of a healthcare system in one country is dysfunctional, it will lead to an unreasonable pharmaceutical price. The data results thus show that a functional cost-compensation mechanism is of great necessity. When the outcome is an unreasonable pharmaceutical price, the *Consistency_~CCS_* is above 0.9. When the outcome becomes a reasonable pharmaceutical price, the *Consistency_CCS_* closely approaches 0.

### Sufficiency analysis of conditional configuration

In contrast to the necessary condition analysis described above, the group analysis attempts to reveal that different conditional configurations lead to diversified results. From a set-theoretic perspective, this means exploring whether the set represented by multiple conditional groupings is a subset of the set of outcomes. The same consistency is used to measure the adequacy of configurations. Consistency is also used to measure the sufficiency of configurations, but the minimum acceptable criteria and calculation methods are different from those of the necessary condition analysis [34]. Schneider and Wagemann stated that the level of consistency needed to find sufficiency must not be less than 0.75 [34]. The determination of the frequency threshold depends on the sample size. For small- or medium-scale samples, a frequency threshold of 1 is sufficient, while for large-scale samples, the frequency threshold should be greater than 1. Based on the above-mentioned analysis, we chose 1 as the frequency threshold as the sample adopted was not a large-scale sample.

We have presented the results of our QCA in the style of Fiss et al. [35]. The advantage of this presentation is that it provides a clear indication of the relative importance of each condition in the configuration. According to their research on QCA, “O” is the core condition (a condition that exists in both the parsimonious and intermediate solutions) and “o” is the auxiliary condition (a condition that exists only in the intermediate solutions). Similarly, “X” is the absence of the core condition, and “x” is the absence of the auxiliary condition. Moreover, “blank space” indicates an ambiguous state, i.e., the condition can be present or absent. These results are provided in Table 5.

**Table 5:** Configuration strongly related to pharmaceutical price.

Antecedent conditions	Unreasonable pharmaceutical price			Reasonable pharmaceutical price	
	config.1	config.2	config.3	config.4	config.5
Induced demand (ID)		X	O	X	
Tiered healthcare system (THS)	X		O		X
Payment mechanism (PM)	X	X	O		O
Cost compensation system (CCS)	X	X	X	O	O
Raw coverage	0.67	0.67	0.11	0.96	0.13
Unique coverage	0.11	0.11	0.11	0.88	0.04
Consistency	1.00	1.00	1.00	0.96	1.00
Overall solution coverage	0.89			1.00	
Overall solution consistency	1.00			0.96	

Note: O represents the core casual condition presence; O represents the peripheral casual condition presence; X represents the core casual condition absence; X represents the peripheral casual condition absence.

According to the results in Table 5, it can be observed that all conditional configurations of antecedents have been proven to explain the mixed causal pathways, for their consistency levels are above 0.75. In the scenario of “unreasonable pharmaceutical price”, there are three main conditional configurations of antecedents. In the scenario of “reasonable pharmaceutical price”, there are two. The explanation of the results is as follows.

On the one hand, in scenario 1 (the outcome is “PP”), configuration 1 reveals that the absence of THS and PM can result in unreasonable pharmaceutical prices (~THC*~PM*~CCS). Moreover, configuration 2 shows that the absence of ID and PM can lead to unreasonable pharmaceutical prices (~ID*~PM*~CCS). Configuration 3 displays how the coexistence of ID, THC and PM and the absence of CCS can lead to unreasonable pharmaceutical prices (ID*THC*PM*~CCS). On the other hand, in scenario 2 (the outcome is “~PP”), two configurations have been provided. The first one is the absence of ID and the existence of CCS, which can lead to reasonable pharmaceutical prices (~ID*CCS). The second is the absence of THS and the coexistence of PM and CCS, which can also result in reasonable pharmaceutical prices (~THC*PM*CCS).

These hybrid causal pathways, produced through QCA, set up our further discussion of the influence pathways of pharmaceutical prices.

## Discussion

This study determined which factors lead to unreasonable pharmaceutical prices. Given the complexity of the research question, this study utilized QCA to analyze the hybrid impact pathways, and different configurations have been offered. In this part of the article, we further discuss these influencing pathways, to add depth to the configurations we have revealed.

### Cost compensation system: The core influence on the pharmaceutical price

From both the intermediate solution, as well as the parsimonious solution, it can be seen that the low government compensation received for hospital costs (~CCS) appears in every combination of the conditional structures. From the above two combinations of conditional structures, it can be seen that the lack of the government’s share of financial compensation for hospitals leads to inflated drug prices. The low share of financial compensation for public hospitals inevitably makes up for their high costs and allows physicians to receive kickbacks through a system of drug-funded care. This distorted cost reimbursement system inevitably leads to inflated pharmaceutical prices.

When it comes to the explanation, this core condition has revealed the features and positions of the healthcare system. According to the economics of healthcare, there are two points here that we must explore. The first point is that one of the functions of public finance itself is to provide public goods and goods with strictly positive externalities to citizens [24]. The government obtains revenue through taxes and other revenue sources and then provides public goods and private goods with strictly positive externalities to its citizens. As a very large positive externality and welfare good, public hospitals are supposed to be provided by the government. The second point is that hospitals have a large number of positive externalities, so the government compensates public hospitals through public finance to help them improve their public welfare and to ensure the fairness of medical services to the public [6]. Moreover, through hospitals, the government can improve the efficiency of disaster relief, which fulfills the government’s responsibility.

Based on the above-mentioned discussion, proposition 1 of this study is as follows.

**Proposition 1. Cost compensation of the healthcare system that relies less on state financial compensation is not beneficial to pharmaceutical prices regulation**.

### Payment mechanism: Need to employ DRGs

As providers of healthcare services, hospitals and doctors have huge operating costs and need material incentives to motivate providers to offer better services, so as the most important incentive for providers, payment systems for healthcare services vary, but the focus remains on the cost and quality of services.

The majority of countries have an FFS healthcare system. The main reason for the popularity of this fee-for-service system in most countries is that it provides a clear incentive for doctors to provide care to more patients in a shorter period. It is also a payment system that reflects the workload of the doctor to a certain extent. However, the drawbacks of this system are obvious, mainly because of the asymmetry of information in the market of medical services, which allows doctors to induce demand from patients, with them consuming more useless medical services.

While there are many ways to pay for healthcare services, the focus of attention remains on the cost and quality of care. In a fee-for-service payment method, there are no corresponding incentives for healthcare cost-savings. Given the information asymmetry in the healthcare market, doctors are inducing too much demand from patients, leading to greater consumption of healthcare services. The excessive consumption of medical services will lead to an increase in the costs of medical service providers, which leads to a waste of medical and health resources. At the same time, in the medical service market, the waste of resources will inevitably lead to a shortage of resources, so it will also raise the prices of medical services, but indirectly. Secondly, there is the payment-by-disease approach (DRGs), which does not provide corresponding incentives for quality. At the end of the day, patients pay for medical services according to the corresponding disease group, so the revenue of the medical service provider is directly limited. To improve their efficiency and gain more revenue, providers will try to divide their patients into case groups with higher consumption and will not provide better-quality services to patients during their treatment. According to the QCA results, proposition 2 of this study can be offered as follows.

**Proposition 2. Diversified payment mechanisms influencing the pharmaceutical price, especially with an absence of DRGs, do not benefit pharmaceutical prices regulation**.

### Tiered healthcare system and induced demand: A comprehensive impact

When it comes to a tiered healthcare system, a large number of countries have employed one, and according to prior studies, this system plays a critical role in healthcare. Yet, there are still some countries without such a healthcare system. To further discuss the function this system exerts on healthcare, in this section, we choose China as a qualified example where induced demand can also be analyzed.

In China’s healthcare market, uneven allocation of resources and serious wastage of resources is a pressing problem. The implementation of tiered diagnosis and treatment will indeed help China to solve this thorny problem in the healthcare market. First of all, this can avoid overcrowding in large tertiary public hospitals. Due to the information asymmetry in the healthcare market, coupled with the large size of public tertiary hospitals, their complete equipment, and the high technical level of their doctors, people tend to go to large public tertiary hospitals when they are sick. However, the number of public tertiary hospitals in a region is limited, and China’s population base is large, which leads to the overuse of public tertiary hospitals.

Secondly, it helps to improve China’s primary medical services. It is the overcrowding of large tertiary care hospitals that has left China’s primary care providers unattended. Even though the service system of primary care providers in China is improving, there are still the following shortcomings: the distribution of primary care resources is unbalanced and there is a big difference between urban and rural areas; the government’s financial compensation system for primary care providers is not sound; primary care providers do not have a comprehensive understanding of their social functions due to their limited level. These deficiencies also exacerbate the unbalanced allocation of resources in China’s healthcare market, further skewing the distribution and seriously wasting resources.

However, with the absence of a tiered healthcare system, it is of great necessity to utilize induced demand. In China, drug-based care is of great popularity. A system of drug-based care can indirectly support physicians’ induced demand behavior. Specifically, government subsidies are relatively low, and hospitals receive relatively low revenues from elsewhere, so they have to cover their costs by selling drugs. In China, public hospitals are usually relatively large, and the revenues through government subsidies can be considered almost a drop in the bucket. Therefore, hospitals have to expand their sales of drugs to make a living. The second point is on the process of prescribing drugs by doctors. Doctors have to get patients to go to the hospital to buy drugs by prescribing them. Based on the benchmark model, to maximize their utility, physicians must maximize their net income. Based on the model analysis of the process of physicians’ prescribing, there is an induced demand from the physician, i.e., the physician will over-prescribe to the patient to make the hospital more profitable.

Based on the above-mentioned examples, it can be easily seen that a tiered healthcare system closely links to induced demand. Moreover, this point of view was also revealed by the results of QCA. Although these two antecedent conditions are auxiliary conditions, we cannot ignore their impact on pharmaceutical prices. Overall, the third proposition of this study may be made as follows.

**Proposition 3. Physician inducements and the absence of a tiered healthcare system are not beneficial to pharmaceutical prices regulation**.

## General discussion

This research emphasizes the relationship between medical service provision and unreasonable pharmaceutical prices. According to the results of this study, medical service exerts a great impact on pharmaceutical prices from the internal perspective, compared with the conventional external perspective. Due to the medical service provision originating from the healthcare system itself, the absence of this research viewpoint has provided a research gap for us. This study aims to fill up this research gap and thus focuses on the impact exerted by the medical service provision on unreasonable pharmaceutical prices.

Specifically, the impact mechanism explored by this study offers theoretical contributions to the related research. First of all, it fills up the gap in the internal research perspective on the unreasonable pharmaceutical price [1,8,20]. The absence of this research perspective results in a shallow understanding of unreasonable pharmaceutical prices, thus leading to anarchy in the regulation of the medical service provision. Not only that, this study extracts the detailed impact mechanism of medical service provision from the macro and micro aspects. The detailed mechanism offers further theoretical contributions in the studies of regulation on pharmaceutical prices. According to a previous study of regulation on pharmaceutical prices, few studies revealed the factors leading to unreasonable pharmaceutical prices from the perspective of medical service provision [7,10,16,35]. This study explores this detailed impact mechanism, which offers several contributions to the regulation of pharmaceutical prices.

Moreover, this study also provides several practical implications. To start with, the proposition in this study gives insights for managers in the healthcare system. When managers want to price drugs appropriately, they can refer to the medical service provision. With a refined analysis of the impact mechanism, administrators can offer reasonable pricing for drugs. Next, this study also provides a basis for government policy. It has been widely recognized that unreasonable pharmaceutical prices may jeopardize the patient’s welfare. Government shall protect the welfare of the patient by regulating the pharmaceutical price from the aspect of medical service provision. Overall, this study offers several theoretical contributions and practical implications for academics and industries.

## Conclusion

According to the above-mentioned discussion, several conclusions can be drawn. Generally speaking, the pharmaceutical price is of great significance for a country’s healthcare system. Ultimately, where the antecedent condition comes from the physician or healthcare system, this problem is closely related to the hospital itself and the institution of the healthcare system.

Specifically, public hospitals have no way to eliminate their own systemic shortcomings, which is why the medical market is chaotic. In the first point, public hospitals are blindly expanding their scale. In some countries, due to the absence of a tiered treatment system, all high-quality resources are concentrated in public hospitals and all patients choose to go to public hospitals for medical treatment. This means that public hospitals face a situation where they can only expand in scale. Several scholars have carried out studies and their conclusions show that the larger the hospital, the less efficient it is (e.g., Frech and Mobley, 1995) [36]. Blindly expanding the size of public hospitals can only lead them to accept the double suppression of inefficiency and high costs. Secondly, public hospitals receive a small share of government financial compensation, meaning they have great positive externalities. To improve, the government should provide this category of goods, and public hospitals should receive financial compensation from the government. Some developing countries have no way to afford a large-scale healthcare system; as a result, the financial investment in medical services is much less than that in developed countries. This inevitably leads to the creation of a drug-based system that directly distorts the behavior of physicians, given that they receive large kickbacks. This, coupled with the inadequate third-party payment system and funding mechanism for medical services, inevitably leads to unreasonable pharmaceutical prices.

## Limitations and future research

This study explored the antecedents of unreasonable pharmaceutical prices. To deal with the complex research question, the study used QCA for its analysis. Based on 33 case countries, a configuration framework was established. At the end of this study, we proposed three propositions to detect the antecedent conditions of pharmaceutical prices. However, there are some limitations. First of all, as a data limitation, 33 countries marks a shortcoming in terms of the sample size. Future studies must include more case countries. Second, the shortcomings of QCA itself have limited us from conducting a more concrete analysis of a single condition. Future studies should overcome this by using different forms of analysis. Third, the antecedents put forward in this study are limited in their scope; future research should expand on these.

## Supporting information

S1 Data (XLSX)
